# Enhancing Survival in Septic Shock: A Systematic Review and Meta-Analysis of the Efficacy of Plasma Exchange Therapy

**DOI:** 10.7759/cureus.60947

**Published:** 2024-05-23

**Authors:** Grethel N Hernandez, Aida J Francis, Pousette Hamid

**Affiliations:** 1 Infectious Diseases, Louisiana State University Health Sciences Center, Shreveport, USA; 2 Internal Medicine, California Institute of Behavioral Neurosciences & Psychology, Fairfield, USA; 3 Family Medicine, California Institute of Behavioral Neurosciences & Psychology, Fairfield, USA; 4 Neurology, California Institute of Behavioral Neurosciences & Psychology, Fairfield, USA

**Keywords:** mortality reduction, septic shock, therapeutic plasma exchange (tpe), plasma exchange therapy, plasmapheresis

## Abstract

Sepsis is a life-threatening condition that occurs when the body’s immune response to infection becomes unregulated, causing organ dysfunction and a heightened risk of mortality. Despite increased awareness campaigns, its prevalence escalates, annually afflicting over 1.7 million adults in the United States. This research explores the potential of therapeutic plasma exchange (TPE) in septic shock management, aiming to highlight its capacity to improve patient outcomes and reduce mortality. Adhering to the Preferred Reporting Items for Systematic Reviews and Meta-Analysis guidelines, our comprehensive search across 51,534 studies, using keywords such as plasmapheresis, plasma exchange therapy, therapeutic plasma exchange, septic shock, and reduction in mortality integrated with medical subject headings terms, led to the meticulous selection of six pivotal studies. Through rigorous evaluation with tools such as the revised Cochrane Risk-of-Bias tool, Newcastle-Ottawa Scale, and Assessment of Methodological Quality of Systematic Reviews, we extracted strong evidence supporting TPE’s significant impact on decreasing mortality in septic shock patients compared to standard care, as demonstrated in three randomized controlled trials and one cohort study, with an odds ratio (OR) of 0.43 (95% confidence interval (CI) = 0.26-0.72). Additionally, two meta-analyses further validate TPE’s effectiveness, showing a mortality reduction with an OR of 0.30 (95% CI = 0.20-0.46). This advantage also extends to critically ill COVID-19 patients, underscoring TPE’s crucial role in modulating the coagulation cascade, decreasing sepsis-related complications, and reducing the risk of bleeding and organ failure. Nevertheless, the benefits of TPE must be carefully balanced against potential risks such as hypocalcemia, hypotension, and citrate toxicity, especially in patients with underlying renal or liver issues, emphasizing the importance of shared decision-making. While TPE emerges as a promising therapy, its formal integration into standard care protocols awaits further confirmation, highlighting the critical need for more in-depth research to conclusively determine its efficacy and safety in septic shock management.

## Introduction and background

Sepsis is a life-threatening medical condition characterized by organ dysfunction resulting from an abnormal response to infection [1]. It progresses to septic shock when it includes severe circulatory, cellular, or metabolic abnormalities that greatly increase the risk of death. Clinical indicators of septic shock include the need for vasopressor therapy to maintain a mean arterial blood pressure of 65 mmHg or higher, as well as lactate levels exceeding 2 mmol/L (18 mg/dL) after adequate fluid resuscitation.

Despite advancements in sepsis campaigns, its incidence continues to surge, affecting over 1.7 million adults in the United States annually [2]. Of those, at least 350,000 succumb to sepsis-related complications or transition to hospice care, representing one in every three hospital deaths. Recent studies, such as Bauer et al., reveal average 30-day septic shock mortality at 34.7% and 90-day mortality at 38.5% [3]. The 2021 Surviving Sepsis Campaign (SSC) guidelines have been updated to stress the importance of early detection and treatment strategies in the care of critically ill patients. These strategies include tools such as quick Sequential Organ Failure Assessment (qSOFA), National Early Warning Score (NEWS), and Modified Early Warning Score (MEWS). However, the guidelines caution against relying solely on qSOFA in the absence of severe inflammatory response syndrome, NEWS, or MEWS criteria, as these tools are all essential in improving patient care [1].

Enhanced education and awareness are crucial, stressing sepsis as a medical emergency [1]. The evolving care bundles, revised with shorter timeframes, highlight the pivotal role of emergency physicians in prompt recognition and immediate initiation of septic patient emergency resuscitation and treatment. Education, ongoing clinical research, and adherence to guidelines are key components aimed at reducing mortality rates.

Extracorporeal blood purification methods have been suggested as additional treatment options for sepsis [4]. These approaches operate under the idea that eliminating or adjusting levels of pro- and anti-inflammatory agents or bacterial toxins in the blood may diminish the overwhelming systemic inflammatory response in sepsis, potentially reducing both the severity of the condition and the likelihood of death.

Therapeutic plasma exchange (TPE) encompasses extracting whole blood from the patient, separating it into components, eliminating the patient’s plasma, and then reintroducing the patient’s other blood components alongside replacement fluids such as 5% albumin or fresh frozen plasma (FFP) [5]. By diminishing plasma components such as auto and alloantibodies, plasma proteins, and inflammatory mediators, this procedure has been suggested as an additional therapy for treating COVID-19.

Therapeutic plasmapheresis, previously used in treating acute respiratory distress syndrome (ARDS) during the 2009 H1N1 influenza pandemic, shows promise in reducing pro-inflammatory cytokines in septic shock patients [6]. It potentially reduces the need for multiple medications targeting various cytokines, thus minimizing polypharmacy-related side effects and dose adjustments for patients with comorbidities.

There is a need for more research on TPE and its effectiveness in reducing mortality and improving outcomes in sepsis treatment. A comprehensive examination of its influence on survival rates and clinical parameters is essential, along with assessing the sustainability of benefits over time, understanding optimal patient selection, and determining the best timing for initiating the therapy. This study aims to evaluate the efficacy of plasma exchange as a therapeutic intervention and shed light on its potential role in enhancing patient prognosis and reducing mortality risk during septic shock episodes.

## Review

Methodology

The current systematic review followed the Preferred Reporting Items for Systematic Reviews and Meta-Analysis (PRISMA) guidelines [7].

Search Strategy

Two investigators (GNH and AF) conducted independent searches on various databases, including PubMed, the Cochrane Central Register of Clinical Trials, ScienceDirect, MDPI (Multidisciplinary Digital Publishing Institute), and Google Scholar from September 6, 2023, to January 5, 2024, to find relevant articles using the following keywords: plasmapheresis, plasma exchange therapy, therapeutic plasma exchange, septic shock, mortality reduction with Boolean AND along with medical subject headings (MeSH) (“Plasma Exchange”[Mesh]) AND (“Shock, Septic/complications”[Mesh] OR “Shock, Septic/mortality”[Mesh]).

Study Selection

Eligible studies needed to fulfill the following Population, Intervention, Comparator, Outcome, and Study design (PICOS) criteria: (a) critically ill adults and children experiencing sepsis, with accompanying septic shock; (b) plasmapheresis or plasma exchange therapy as intervention; (c) guideline-directed therapy for septic shock as control group; (d) outcome of reduction in mortality; (e) open-access randomized controlled trials (RCTs), cohort studies, and systematic reviews and meta-analyses published within the last five years.

The study excluded other blood purification techniques, plasmapheresis use in other medical conditions, case reports, and case series. Two authors (GNH and AF) evaluated selected studies separately, excluding those lacking mortality data or full-text articles. No language restrictions were imposed.

Selection Process

The authors (GNH and AF) independently assessed the internal validity of each study using various tools, including the Revised Cochrane Risk-of-Bias tool for randomized trials (RoB2) [8], the Newcastle-Ottawa quality assessment for cohort studies [9], and the Assessment of Methodological Quality of Systematic Reviews (AMSTAR) for systematic reviews [10]. Each RCT was evaluated for bias, receiving ratings of “Low,” “High,” or “Some concerns.” Table 1 displays the quality assessment tool findings for RCTs.

**Table 1:** Summary of quality assessment for randomized controlled trials.

	Bias arising from the randomization process	Bias due to deviations from the intended intervention	Bias due to missing outcome data	Bias in the measurement of the outcome	Bias in the selection of the reported result
Weng et al. (2021) [11]	Low	Low	Low	Low	Low
Faqihi et al. (2021) [12]	Some concerns	Some concerns	Low	Some concerns	Some concerns
Stahl et al. (2022) [13]	Some concerns	Some concerns	Low	Some concerns	Some concerns

In evaluating the quality of a retrospective cohort study, we employed the Newcastle-Ottawa tool [9]. A summary of the assessment is provided in Table 2.

**Table 2:** Newcastle-Ottawa assessment tool for cohort study.

Study	Study design	Selection	Comparability	Outcome	Total
Keith et al. (2020) [14]	Retrospective cohort	****	*	***	8

For the assessment of systematic review quality, we utilized the AMSTAR checklist [10] presented in Table 3.

**Table 3:** Summary of quality assessment for systematic reviews and meta-analyses. AMSTAR = Assessment of Methodological Quality of Systematic Reviews

AMSTAR 2 checklist	Putzu et al. (2021) [4]	Qin et al. (2022) [15]
Q1: Did the research questions and inclusion criteria for the review include the components of PICO?	Yes	Yes
Q2: Did the report of the review contain an explicit statement that review methods were established before the conduct of review and did the report justify any significant deviations from the protocol?	Yes	Yes
Q3: Did the review authors explain their selection of the study designs for inclusion in the review?	No	No
Q4: Did the review authors use a comprehensive literature search strategy?	Partial Yes	Yes
Q5: Did the review authors perform study selection in duplicate?	Yes	No
Q6: Did the review authors perform data extraction in duplicate?	Yes	Yes
Q7: Did the review authors provide a list of excluded studies and justify the exclusions?	Yes	No
Q8: Did the review authors describe the included studies in adequate detail?	Yes	No
Q9: Did the review authors use a satisfactory technique for assessing the risk of bias in individual studies that were included in the review?	No	No
Q10: Did the review authors report on the sources of funding for the studies included in the review?	Yes	Yes
Q11: If a meta-analysis was performed, did the review authors use appropriate methods for statistical combinations of results?	Yes	Yes
Q12: If a meta-analysis was performed, did the review authors assess the potential impact of risk of bias in individual studies on the results of the meta-analysis or other evidence synthesis?	Yes	No
Q13: Did the review authors account for the risk of bias in individual studies when interpreting/discussing the results of the review?	Yes	No
Q14: Did the review authors provide a satisfactory explanation for and discussion of any heterogeneity observed in the results of the review?	Yes	No
Q15: If they performed quantitative synthesis, did the review authors carry out an adequate investigation of publication bias and discuss its likely impact on the results of the review?	No	No
Q16: Did the review authors report any potential sources of conflict of interest, including any funding they received for conducting the review?	Yes	Yes

Statistical Analysis

A meta-analysis using RevMan 5.4.1 software evaluated the relationship between therapeutic plasmapheresis and mortality reduction. Pooled odds ratios (ORs) and 95% confidence intervals (CIs) were calculated using the Mantel-Haenszel method under a fixed-effect model. Forest plots were generated to visually display the results. Tests for heterogeneity and overall effect were conducted using the chi-square test and z-score.

Results

Study Identification and Selection

**Figure 1:**
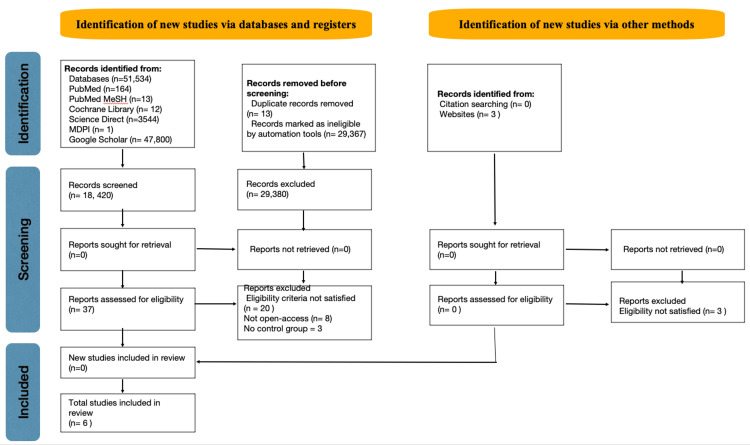
Summary of PRISMA flowchart. MDPI = Multidisciplinary Digital Publishing Institute; PRISMA = Preferred Reporting Items for Systematic Reviews and Meta-Analysis

The search strategy initially identified 51,534 studies across five databases. After filtering, 18,420 were manually screened using the PICOS criteria, and 13 duplicate articles were excluded. There were 37 articles gathered through abstract scanning and six studies were evaluated using quality assessment tools for inclusion in the review. The summarized findings are presented in Figure 1.

There were three RCTs [11-13], two systematic review and meta-analysis studies [4,15], and one retrospective cohort [14] were included in the review.

A study by Weng et al. found that therapeutic plasmapheresis is more effective than heparin in improving platelet count, coagulation function, and reducing bleeding events, acute kidney injury (AKI), and ARDS in patients with sepsis-associated disseminated intravascular coagulation (DIC), suggesting therapeutic plasmapheresis could be a more efficient therapy [11].

A clinical trial by Faqihi et al. found that plasma exchange therapy improved clinical outcomes in intensive care unit (ICU) patients with severe COVID-19 [12]. TPE led to quicker recovery, reduced time on mechanical ventilation, and improved biomarkers. The 35-day mortality rate was slightly lower in TPE patients, suggesting that adding TPE to standard ICU therapy may benefit critically ill COVID-19 patients.

The RCT by Stahl et al. on septic shock patients found that TPE led to a consistent decrease in norepinephrine doses, serum lactate levels, biomarkers, and disease mediators, while the standard of care did not [13]. The initial lactate levels could predict individual response to TPE.

A study by Keith et al. compared plasmapheresis to standard therapy in patients with septic shock and multiple organ failure [14]. The observational study between 2015 and 2019 found that plasma exchange therapy reduced mortality, improved SOFA scores, and improved fluid balance. Patients who received additional plasma exchange therapy had longer stays in the ICU and hospital. Although not universally recommended, the study offers valuable insights for future clinical trials.

Putzu et al. reviewed randomized trials on blood purification techniques for sepsis and septic shock, finding that hemoperfusion, hemofiltration, or plasmapheresis can reduce mortality [4]. Still, polymyxin B hemoperfusion does not show a difference, requiring high-quality trials for widespread use.

A study by Qin et al. found that TPE significantly reduced mortality in COVID-19 patients, indicating that plasma exchange therapy should be considered for hospitalized patients with moderate-to-critical COVID-19 [15]. Table 4 provides a summary of the included studies.

**Table 4:** Summary of the characteristics of the included studies. DIC = disseminated intravascular coagulation; HP = heparin; RC = retrospective cohort; RCT = randomized controlled trial; SOC = standard of care; SR = systematic review; MA = meta-analysis; TPE = therapeutic plasma exchange

	Type of study	Sample size (n)	Population	Patient groups	Mortality
Keith et al (2020) [14]	RC	80 (40 TPE, 40 SOC)	Patients with a primary diagnosis of shock and any of the following flags were screened: two or more vasopressors, lactic acid >2 mmol/L, platelet nadir <200 × 10^3^/μL, and pH <7.3	1. TPE. 2. SOC	The mortality rate within 28 days was 40% for the group receiving the intervention and 65% for the control group, showing a significant statistical difference
Weng et al. (2021) [11]	RCT	112 (40 in the TPE group, 36 in the HP group, and 36 in the control group)	Sepsis-related DIC patients	1. Control group (conventional treatment). 2. HP group (low-dose HP anticoagulant). 3. TPE group (conventional + TPE)	The 28-day mortality rate was lower in the TPE group compared to the control and HP groups
Putzu et al. (2021) [4]	SR and MA	128 patients, two trials under plasmapheresis	Sepsis, severe sepsis, and septic shock	1. Polymyxin B immobilized fiber column hemoperfusion. 2. Hemoperfusion with other devices. 3. Hemofiltration techniques. 4. Combined hemofiltration and hemoperfusion techniques. 5. Plasmapheresis techniques	A subsequent RCT with a larger sample size found a 20.5% reduction in absolute mortality risk. Although the results are promising, there is insufficient evidence to recommend plasmapheresis for blood purification in sepsis
Faqihi et al. (2021) [12]	Single-center, open-label RCT	87 (43 in the intervention group, 40 in the control group)	Critically-ill COVID-19 patients	1. Control group (standard treatment for COVID-19). 2. Intervention group (standard treatment + TPE)	Kaplan-Meier analysis revealed no statistically significant difference in the average survival distribution of patients in the TPE group versus the control group
Stahl et al. (2022) [13]	Open-label bicenter RCT	40 (20 SOC, 20 SOC + TPE)	Septic shock lasting less than 24 hours and requiring at least 0.4 μg/kg/minute of norepinephrine	1. SOC. 2. SOC + TPE	Within 48 hours, the rate of death was 30% in the SOC group compared to 10% in the TPE group following randomization
Qin et al. (2022) [15]	SR and MA	343 (173 in the TPE group, 170 in the control group)	Severe-to-critical, moderate-to-critical, critical, and severe	1. Plasma exchange group. 2. Control group	The mortality rate was significantly lower in the TPE groups compared to the SOC

Additional attributes of the included studies such as gender, age, comorbidities, SOFA, and Acute Physiology and Chronic Health Evaluation II (APACHE) scores are included in Table 5.

**Table 5:** Additional attributes of the study groups. APACHE II = Acute Physiology and Chronic Health Evaluation II; CAD = coronary artery disease; CHF = congestive heart failure; CKD = chronic kidney disease; COPD = chronic obstructive pulmonary disease; DM = diabetes mellitus; HSCT = hematopoietic stem cell transplant; SOC = standard of care; SOFA = Sequential Organ Failure Assessment; SOT = solid organ transplant; TPE = therapeutic plasma exchange

	Gender (M)	Mean age (years)	Comorbidities	Mean SOFA score (baseline)	Mean APACHE II score
Keith et al (2020) [14]	TPE 24/40. SOC 21/40	TPE: 57.6 ± 13.4. SOC: 63.6 ± 16.3	Hypertension, CKD, DM, COPD	TPE 14.3 ± 3.6. SOC 13.8 ± 2.4	TPE 32.5 ± 6.0. SOC 32.7 ± 7.2
Weng et al. (2021) [11]	TPE 31/40. Heparin 28/36. Sham 27/36	TPE 50.07 ± 2.09. Heparin 47.92 ± 2.58. Sham 49.73 ± 2.50	None	TPE 11 ± 1.31. Heparin 10.61 ± 1.39. Sham 11.09 ± 1.41	TPE: 20.29 ± 1.29. Heparin 19.17 ± 1.69. Sham 21.09 ± 1.73
Putzu et al. (2021) [4]	None	None	None	None	None
Faqihi et al. (2021) [12]	TPE 36/43. Control 36/44	TPE 48.3. Control 49	Hypertension, DM, CAD	TPE 10 (8–13). Control 9 (6–12)	TPE 23 (21–25). Control 22 (21–23)
Stahl et al. (2022) [13]	TPE 15/20. SOC 17/20	TPE 55. SOC 57	Obesity, Hypertension, DM, CHF, CAD, CKD, SOT, HSCT	TPE 16 (13–18). SOC 18 (14–20)	None
Qin et al. (2022) [15]	40–100%	48–64	DM, hypertension	3 - 10	5.7 - 23

Mortality in Septic Shock: Therapeutic Plasma Exchange Compared to Standard of Care

Mortality information was obtained from multiple sources, including three RCTs, two systematic reviews and meta-analyses, and one retrospective cohort study. The data showed that the mortality rate was significantly lower in groups that underwent TPE compared to those who received standard of care.

**Figure 2:**
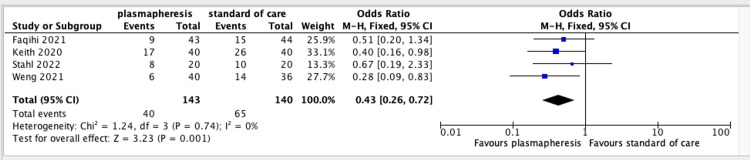
Forest plot assessing the efficacy of therapeutic plasma exchange on reduction of mortality using the included studies in the review. CI = confidence interval; M-H = Mantel-Haenszel

Four studies [11-14] showed that plasmapheresis is more effective than standard of care in reducing mortality (OR = 0.43; 95% CI = 0.26-0.72). The chi-square statistic and I² statistic indicated no significant heterogeneity between studies, and the Z-score of 3.23 confirmed its effectiveness. The forest plot showed consistent effect sizes across studies and a statistically significant overall effect in favor of plasmapheresis. Figure 2 displays a forest plot of three RCTs and one cohort study assessing the efficacy of plasmapheresis and its influence on mortality.

**Figure 3:**
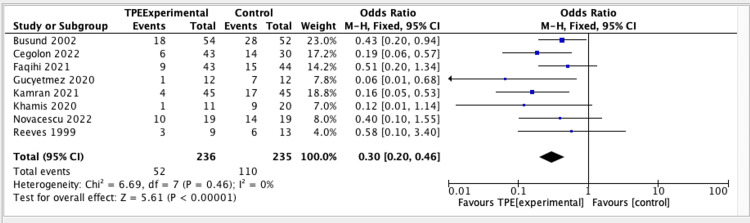
Forest plot assessing the efficacy of TPE on mortality based on the systematic review and meta-analysis of Putzu et al. and Qin et al. CI = confidence interval; M-H = Mantel-Haenszel; TPE = therapeutic plasma exchange

A systematic review and meta-analysis by Putzu et al. and Qin et al. found no significant heterogeneity among eight RCTs assessing the efficacy of plasmapheresis on mortality rate. The results showed a statistically significant overall effect of TPE compared to control or alternative interventions (OR = 0.30; 95% CI = 0.20-0.46), with a p-value of less than 0.00001, indicating strong evidence against the null hypothesis of no effect. The absence of heterogeneity indicates uniform effect sizes throughout the studies, enhancing the reliability of the results. The forest plot in Figure 3 supports the conclusion that TPE has a significant beneficial effect on mortality rate.

**Figure 4:**
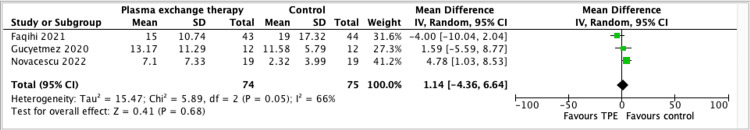
Forest plot assessing the efficacy of TPE on the duration of invasive mechanical ventilation in critically ill COVID-19 patients based on the data gathered in the systematic review and meta-analysis of Qin et al. CI = confidence interval; M-H = Mantel-Haenszel; SD = standard deviation; TPE = therapeutic plasma exchange

The systematic review and meta-analysis by Qin et al. found that therapeutic plasmapheresis may not significantly reduce invasive mechanical ventilation duration in critically ill COVID-19 patients. However, the results should be interpreted cautiously due to heterogeneity between studies and lack of statistical significance. The control group had a slight edge, suggesting uncertainty in TPE’s impact. The forest plot in Figure 4 suggests that TPE might not significantly reduce the duration of invasive mechanical ventilation in these patients.

Discussion

We undertook a comprehensive review and meta-analysis of multiple studies to assess the impact of therapeutic plasmapheresis on mortality rates in patients suffering from septic shock. By aggregating and synthesizing data from these varied studies, we aimed to achieve a clearer understanding of how effective plasmapheresis is as a treatment option. This involved critically evaluating the methodologies and findings of each study, comparing their outcomes, and statistically analyzing pooled data to determine whether this treatment significantly improves survival rates among these critically ill patients. The goal was to provide a robust evidence base to guide clinical decision-making in the management of septic shock.

Pathophysiology of Septic Shock

When the innate immune cells recognize pathogens, they release inflammatory mediators that activate coagulation and cause vasodilation and endothelial leakage, leading to organ dysfunction and hypotension in sepsis [16]. The inflammatory response also triggers the production of procoagulant factors while decreasing natural anticoagulant factors, resulting in a procoagulant state characterized by multiple microthrombi that obstruct small vessels, leading to DIC.

**Figure 5:**
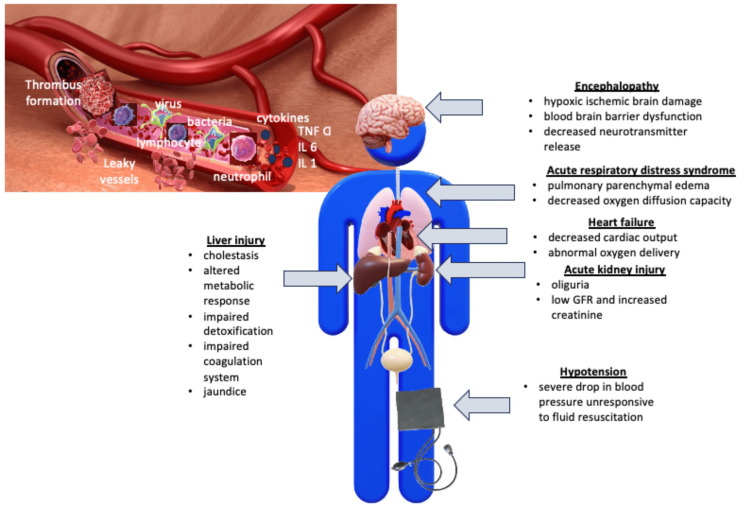
Septic shock pathophysiology: impaired endothelial function causing vascular leakage and thrombosis leading to multiple organ dysfunction. IL = Interleukin; TNF-α = tumor necrosis factor-alpha The figure was created by the corresponding author GNH and co-author AF.

In sepsis-associated DIC patients, thrombocytopenia occurs due to toxins produced by the infection, excessive platelet consumption, and the production of a large number of autoimmune antibodies [11]. These patients have impaired endothelial function and damage, leading to coagulopathy and mortality. Figure 5 shows how septic shock can cause thrombosis in various organs and its complications.

Treatment Guidelines in Sepsis

In a 2018 update, the SSC guidelines introduced an Hour-1 bundle, merging the previously separate three-hour and six-hour care bundles for sepsis [1]. This revised package, designed to start treatment for sepsis patients right away, includes the following five essential actions: checking serum lactate levels and checking again in two to four hours if they exceed 2 mmol/L, taking blood cultures before administering antibiotics, giving broad-spectrum antibiotics, administering 30 mL/kg fluid resuscitation for hypotension or lactate levels over 4 mmol/L, and using vasopressors to sustain a mean arterial pressure over 65 mmHg if hypotension persists during or after fluid resuscitation. The objective is to fulfill these steps within the first hour of diagnosing sepsis, either at triage or upon meeting the sepsis criteria.

Furthermore, the American Society for Apheresis (ASFA) has rated TPE in the management of septic shock with multiorgan failure as Category III, with a Grade 2B recommendation [17]. This classification implies that the use of TPE in treating septic shock with multiorgan failure is uncertain, falling into Category III. This suggests conflicting evidence or varying opinions about its effectiveness or utility, making it not strong enough to be a standard treatment. However, it may be considered an alternative option, particularly in specific situations where benefits and risks are carefully evaluated.

Plasma Exchange Therapy

Plasma exchange therapy is a method used to remove pathogenic substances from plasma, with different flow rates and setups [17]. It can be continuous or intermittent, and needle sizes vary between adults and children. Blood is withdrawn from larger veins and replaced in smaller ones. Ports connect major veins, and citrate is the preferred anticoagulant due to its regional effect and safety. Plasma exchange replenishes components such as A disintegrin and metalloprotease with thrombospondin-1-like domains 13 (ADAMTS13) and clotting factors in bleeding patients. The selection of replacement fluid for apheresis is guided by the purpose of the procedure, alongside considerations of infection and bleeding risks. Typically, the plasma used is either FFP or plasma frozen within 24 hours.

**Figure 6:**
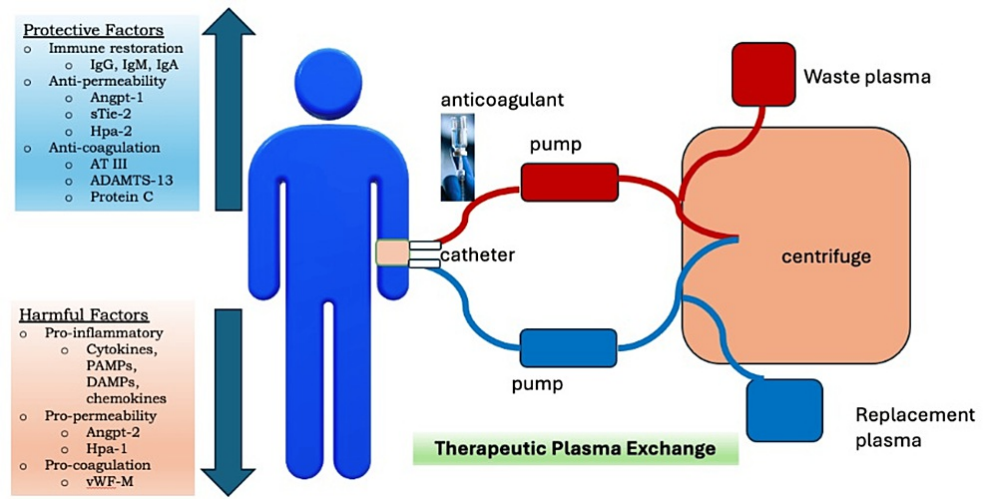
How therapeutic plasma exchange works in sepsis. Plasma exchange therapy increases protective factors and reduces harmful factors during sepsis and septic shock. ADAMTS-13 = A disintegrin and metalloprotease with thrombospondin-1-like domains 13; Angpt-1 = angiopoietin-1; Angpt-2 = angiopoietin-2; AT-III = anti-thrombin-III; DAMPs = damage-associated molecular patterns; Hpa-1 = heparanase-1; Hpa-2 = heparanase-2; IgA = immunoglobulin A; IgG = immunoglobulin G; IgM = immunoglobulin M; PAMPs = pathogen-associated molecular patterns; sTie-2 = soluble receptor for tyrosine kinase with immunoglobulin-like and endothelial growth factor-like domains 2; vWF-M = (ultra) large von Willebrand factor multimers The figure was created by the corresponding author GNH.

Stahl et al. have suggested that therapeutic plasmapheresis has the potential to not only eliminate excessive harmful mediators but also replenish depleted protective factors found in healthy donor plasma [13]. In their study, they observed a reduction in harmful mediators such as procalcitonin, von Willebrand factor antigen, angiopoietin-2, and soluble receptor for tyrosine kinase with immunoglobulin-like and endothelial growth factor-like domains-2, and restoration of decreased protective factors such as anti-thrombin-III, Protein C, and ADAMTS-13, which were not observed in the control group. Figure 6 illustrates the mechanism of therapeutic plasmapheresis in the context of sepsis.

Recent Studies About Therapeutic Plasma Exchange

Our meta-analysis, incorporating three RCTs, one cohort study, and an additional eight RCTs sourced from meta-analyses conducted by Putzu et al. and Qin et al., revealed that plasma exchange therapy significantly reduces mortality among septic shock patients compared to standard medical therapy alone. This benefit extends to critically ill COVID-19 patients as well.

Plasma exchange therapy has been shown to have a positive impact on blood coagulation and platelet count, which can reduce the risk of bleeding events in individuals with sepsis-related DIC [11]. Therefore, incorporating TPE alongside conventional treatment can potentially improve the overall health and recovery outcomes of such individuals. In a study conducted by Weng et al., TPE was found to be more effective than heparin in increasing platelet count, improving coagulation function, increasing the 28-day cumulative survival rate, and reducing the length of ICU hospitalization, 28-day mortality, as well as the incidence of bleeding events, AKI, and ARDS. Moreover, the effect of TPE was superior to that of heparin on endothelial function in sepsis-associated DIC patients.

A study conducted by Faqihi et al. among patients with severe COVID-19 found that adding TPE to standard therapy resulted in clinical improvement [12]. However, it did not significantly impact the 35-day mortality rate. Observations showed a notable decrease in SOFA scores in the TPE group compared to the control group after 7 and 14 days of therapy initiation, indicating better organ function.

Keith et al. found that adjunct therapeutic plasmapheresis can be beneficial and play a role in treating sepsis with multiple organ failure [14]. The observed 25% absolute decrease in mortality was statistically significant and strongly suggested a clinical advantage. Although the study reported a high overall mortality rate, this aligns with past figures once adjusted for severity of illness (73-95.2%, considering admission APACHE II and SOFA scores).

Conversely, a secondary analysis by Luo et al. of a subset of 742 septic patients from a cohort of 2,772 patients found no significant differences in delta SOFA scores or 28-day mortality rates between patients who received TPE and control patients [18]. Interestingly, the TPE group experienced significantly fewer ICU-free and alive days, suggesting that TPE might not improve outcomes related to organ failure or mortality in critically ill sepsis patients and could lead to prolonged ICU stays.

Zhang et al. presented a contrasting view, identifying plasmapheresis as associated with longer ICU stays but also with a substantial reduction in all-cause mortality among adults [19]. Despite mixed outcomes across different age groups, the overall benefits of plasmapheresis are underscored by a notable reduction in mortality, with minimal adverse events reported.

In a large-scale review by Lee et al. involving over 50,000 patients, adults with severe sepsis who underwent TPE with FFP showed lower mortality rates [20]. However, in septic children without thrombocytopenia-associated multiorgan failure, TPE was linked to higher mortality. The study also highlighted that different TPE techniques (centrifugal vs. membrane) did not significantly affect outcomes, but continuous TPE regimens negatively impacted both adults and children.

Further research into the efficacy of TPE in sepsis is ongoing, exemplified by the EXCHANGE-2 study by David et al. [21]. This randomized, prospective, multicenter trial focused on early and refractory septic shock patients receiving TPE with donor FFP within six hours of randomization. With a target enrollment of 137 patients per group, the study aimed to demonstrate a 15% improvement in 28-day mortality. Secondary endpoints included SOFA scores and organ support-free days up to day 28, with rigorous monitoring of safety measures such as bleeding, allergic reactions, lung injury, and severe thrombocytopenia.

These findings collectively suggest that while TPE holds promise in certain sepsis populations, its benefits may vary based on patient characteristics and the specific protocols employed.

Adverse Effects of Plasma Exchange Therapy

Plasma exchange therapy is generally safe, but the practitioner must be aware of numerous potential complications. The most common complication is hypocalcemia, which occurs more frequently with frozen plasma (20%) than with albumin (9%) [17]. Patients with reduced citrate excretion in the kidney or liver are more likely to develop citrate toxicity. Hypotension has been reported in 0.4-15% of treatments, and it is more common with albumin-saline solution replacement. Potential mechanisms include delayed or inadequate volume replacement, vasovagal episodes, insufficient oncotic fluid replacement, anaphylaxis, transfusion-associated lung injury, arrhythmia, bradykinin reactions, vascular access bleeding, and cardiovascular collapse. Apheresis has a low mortality rate (0.03-0.05%), and the cause of death is usually due to the underlying disease.

Limitations of the study

Existing studies had insufficient data regarding clinical parameters such as SOFA scores and APACHE II scores. Therefore, the standard deviation could not be calculated to generate a forest plot evaluating the effectiveness of TPE in enhancing these clinical parameters. In addition, most included studies were small and had an unclear or high risk of bias which can potentially undermine the quality and reliability of research findings. Moreover, there were few studies regarding the optimal timing, duration, and number of sessions needed for using TPE to maximize its benefits in septic shock patients with multiorgan failure and DIC.

## Conclusions

The findings from this study underscore the effectiveness of TPE in managing septic shock, providing statistically significant data to support its role in enhancing prognosis and reducing mortality in sepsis patients. TPE appears to improve patient outcomes by modulating the coagulation cascade, alleviating complications such as DIC, and, importantly, minimizing the risks of hemorrhage and organ failure. Despite these benefits, it is crucial to consider the potential adverse effects associated with TPE, such as hypocalcemia, hypotension, and citrate toxicity, especially in patients with underlying renal and liver dysfunction.

The ASFA categorizes TPE as Category III, Grade 2B for septic shock with multiorgan failure, indicating its potential yet not fully established role in standard treatment protocols. This categorization suggests that while TPE can be considered in certain clinical situations where its benefits may outweigh the associated risks, its application requires careful, individualized decision-making that considers the specific conditions of the patient and the expert judgment of the healthcare team. To further define and optimize the use of TPE in sepsis treatment, future research, particularly through large-scale RCTs, should focus on determining the most effective timing, duration, and frequency of TPE sessions to maximize clinical benefits.
